# Atorvastatin Therapy Modulates Telomerase Activity in Patients Free of Atherosclerotic Cardiovascular Diseases

**DOI:** 10.3389/fphar.2016.00347

**Published:** 2016-09-30

**Authors:** Irina D. Strazhesko, Olga N. Tkacheva, Dariga U. Akasheva, Ekaterina N. Dudinskaya, Ekaterina V. Plokhova, Valentina S. Pykhtina, Anna S. Kruglikova, Natalia V. Kokshagina, Natalia V. Sharashkina, Mikhail V. Agaltsov, Daria A. Kashtanova, Vladimir A. Vygodin, Irina N. Ozerova, Dmitry A. Skvortsov, Daria Vasilkova, Sergey A. Boytsov

**Affiliations:** ^1^Department of Aging and Age-Associated Diseases Prevention, National Research Center for Preventive MedicineMoscow, Russia; ^2^Russian Clinical Research Center for GerontologyMoscow, Russia; ^3^Laboratory of Biostatistics, Department of Epidemiology of Chronic Non-Communicable Diseases, National Research Center for Preventive MedicineMoscow, Russia; ^4^Department of Biochemical Markers of Chronic Non-Communicable Diseases Research, National Research Center for Preventive MedicineMoscow, Russia; ^5^Department of Chemistry, Lomonosov Moscow State UniversityMoscow, Russia; ^6^Department of Clinical Cardiology and Molecular Genetics, National Research Center for Preventive MedicineMoscow, Russia

**Keywords:** telomerase activity, chronic inflammation, blood urea, statins

## Abstract

**Background:** Telomerase activity (TA) is considered as the biomarker for cardiovascular aging and cardiovascular diseases (CVDs). Recent studies suggest a link between statins and telomere biology that may be explained by anti-inflammatory actions of statins and their positive effect on TA. Until now, this effect has not been investigated in prospective randomized studies. We hypothesized that 12 months of atorvastatin therapy increased TA in peripheral blood mononuclear cells.

**Methods:** In a randomized, placebo-controlled study 100 hypercholesterolemic patients, aged 35–75 years, free of known CVDs and diabetes mellitus type 2 received 20 mg of atorvastatin daily or placebo for 12 months. TA was measured by quantitative polymerase chain reaction.

**Results:** At study end, 82 patients had sufficient peripheral blood mononuclear cells needed for longitudinal analysis. TA expressed as natural logarithms changed from 0.46 ± 0.05 to 0.68 ± 0.06 (*p* = 0.004) in the atorvastatin group and from 0.67 ± 0.06 to 0.60 ± 0.07 (*p* = 0.477) in the control group. In multiple regression analysis, atorvastatin therapy was the only independent predictor (*p* = 0.05) of the changes in TA independently of markers of chronic inflammation and oxidative stress. Atorvastatin therapy was associated with increases in interleukin-6 within the normal range and a tendency toward reduction in blood urea.

**Conclusion:** These initial observations suggest atorvastatin can act as telomerase activator and potentially as effective geroprotector.

**Trial registration:** The trial was registered in ISRCTN registry ISRCTN55050065.

## Introduction

It is known that statins prolong lifespan in lower organisms (Spindler et al., 2012) and reduce total human mortality, even in people who have normal lipid levels; statins are the first-line drugs in the primary and secondary prevention of cardiovascular diseases (CVDs; Taylor et al., 2011). In many respects, this may be explained by the pleiotropic effects of statins, i.e., which are not associated with lipid-lowering effects. Pleiotropic effects are observed in various cell types including endothelial cells, leukocytes, fibroblasts, and smooth muscle cells (Olivieri et al., 2012). The most widely studied pleiotropic effects of statins are: decreasing of the vascular wall inflammation (Bu et al., 2011), reduced production of intracellular reactive oxygen species (Hong et al., 2006), increasing of the nitric oxide bioavailability (Ni et al., 2001), reduction of platelet aggregation (Casani et al., 2005), angiogenesis stimulation (Shen et al., 2011), immune response modulation (Kwak et al., 2000), and reduction of erythrocyte membranes cholesterol concentration—a marker of clinical instability in patients with coronary heart disease (CHD; Tziakas et al., 2009). The most recently revealed pleiotropic effects of atorvastatin include the prevention of cell aging, the reduction of apoptosis in endothelial progenitor cells, mature endothelial cells, and smooth muscle cells (Assmus et al., 2003; Satoh et al., 2009). Telomere shortening plays a major role in the development of replicative cellular senescence. Telomeres are the regions of repetitive TTAGGG nucleotide sequences at the ends of the linear chromosomal DNA. Shelterin complex, represented by several proteins (TRF1, TRF2, POT1, TIN2, TPP1, Rap1), forms a protective “cap” at the 3′-end of telomere. It protects the linear chromosome ends from degradation and fusion, and is involved in the maintenance of genome stability. Each round of chromosomal replication leads to the telomere shortening. Once the length of telomere DNA becomes extremely short, cell aging occurs, i.e., the cells inability to further divide and to repair damaged DNA (while maintaining the metabolic activity). Telomere dysfunction with similar effects is developing, while removing “shelterin protection.” The increase in the population of old (senescent) cells in tissues reduces the functional capacity of these tissues and the phenotype of aging begins to form. The current data suggest a connection between the processes of telomere shortening, vascular aging, and the development of CVD (Rotar et al., 2012).

Telomerase, which completes telomere DNA repeats, maintains telomere length (TL). Telomerase is a ribonucleoprotein complex that belongs to the DNA-dependent RNA polymerases family (reverse transcriptases). It includes telomerase reverse transcriptase (TERT) and telomerase RNA component (TERC) used as a template for the telomeric DNA synthesis. High telomerase activity (TA) is observed in embryonic stem cells, cancer cells, and human germ cells throughout human life. In cells, in which differentiation is completed, TA decreases and the cells’ telomeres begin to shorten with each division, i.e., each division of these cells leads to aging. This is the typical pattern for most eukaryotic cells. However, there are rare but important exceptions. TA is detected in “mortal” cells such as macrophages and leukocytes.

One of the newly discovered pleiotropic effects of statins is their ability to prevent telomere shortening, both directly and by maintaining the shelterin complex stability and by increasing TA (Brouilette et al., 2007; Satoh et al., 2009; Saliques et al., 2011; Boccardi et al., 2013a). However, there are very few clinical studies which have evaluated the effects of statin therapy on TA. The main goal of our work was to study the effect of atorvastatin on the TA in patients with hypercholesterolemia and without clinical signs of CVD.

Moreover, considering the well-known anti-inflammatory and antioxidant properties of statins, it seems important to assess their impact on major markers of inflammation such as interleukin-6 (IL-6) and the “new” marker of oxidative stress—blood urea (D’Apolito et al., 2015). Our interest in this area was heightened by the recently published data on the controversial effects of IL-6 in the basic cellular processes; specifically, not only the proinflammatory effects of IL-6 but also its anti-apoptotic and anti-inflammatory properties (Scheller et al., 2011) and its essential role in TA regulation (Yamagiwa et al., 2006).

## Materials and Methods

Patients who had passed a preventive outpatient examination in the National Research Center for Preventive Medicine, Moscow, Russia, in 2012–2013 were included in the study. The inclusion criteria were as follows: diagnosed with hypercholesterolemia: low-density lipoprotein cholesterol (LDL-C) ≥160 mg/dL (4.16 mmol/L) in the presence of 0–1 CVD risk factors and LDL-C ≥ 130 mg/dL (3.38 mmol/L) in the presence of two or more CVD risk factors (Grundy et al., 2004); and the absence of any lipid-lowering therapy at the time of inclusion.

Criteria for exclusion from the study were as follows: any chronic somatic diseases, including CVD associated with atherosclerosis, grade 3 hypertension, chronic heart failure [New York Heart Association (NYHA) functional classification III–IV], chronic kidney disease (glomerular filtration rate <60 mL/min/1.73m^2^), liver disease, type 2 diabetes mellitus, acute and chronic inflammatory diseases, cancer, pregnancy, lactation, and refusal to participate in the study. All patients signed a legal informed consent form to participate in the study. The Independent Ethics Committee of the National Research Center for Preventive Medicine, Moscow, Russia approved the study protocol.

Patients visited the clinic to undergo the study protocol examinations from 08:00 to 09:00 AM after a 12-h period of fasting. Blood pressure was measured by using brachial cuff (HEM-7200 M3, Omron Healthcare, Kyoto, Japan) on the right hand in a sitting position three times for 2-min intervals after a 10-min rest; the average of the three measurements was used for analysis. Hypertension was diagnosed when BP was ≥140/90 mm Hg.

The concentrations of total cholesterol, triglycerides, apolipoprotein A1 (ApoA1), and apolipoprotein B (ApoB) were determined by the biochemical analyzer SAPPHIRE-400 using enzyme kits. The concentration of high-density lipoprotein cholesterol was determined by using the same analyzer in the supernatant after precipitation of serum ApoB-containing lipoproteins. The level of LDL-C was calculated by the Friedewald equation (when triglycerides level was no higher than 4.5 mmol/L).The level of the blood urea was measured by enzymatic photometric method on SAPPHIRE-400 analyzer.

The C-reactive protein (CRP) concentration (reference values 0–5 mg/L) and fibrinogen (FBG) concentration (reference values 2–4 g/L) were measured by a highly sensitive immunoturbidimetric method using carboxylated polystyrene particles on a biochemical analyzer, “Sapphire.”

IL-6 level (reference values of 0–10 pg/mL) was measured by using the immunoenzyme method. Malondialdehyde was measured by high-performance liquid chromatography (reference values of 2.2–4.8 mmol/L).

TA was measured using the method described by Kim et al. (1994). The analysis of cellular extract from monocyte fraction of white blood cells (erythrocytes prevents impurity analysis) containing 2 μg of total protein was performed. The cells, derived from the monocytic ring on Ficoll density gradient and washed with phosphate buffered saline (PBS), were re-suspended in lytic buffer [10 mM Tris–HCl and 10 mM 4-(2-hydroxyethyl)-1-piperazineethanesulfonic acid (HEPES)-KOH, pH 7.5, 1.0 mM MgCl_2_, 1 mM ethylene glycol-bis(β-aminoethyl ether)-N,N,N′,N′-tetraacetic acid (EGTA), 5 mM β-mercaptoethanol, 5% glycerol, 0.5% 3-[(3-cholamidopropyl)dimethylammonio]-1-propanesulfonate (CHAPS), 0.1 mM phenylmethylsulfonyl fluoride (PMSF)]. The cells were incubated for 30 min on ice, centrifuged for 10 min at 4°C for 15,000 *g*, and the supernatant solution was collected. The extract was aliquoted and frozen in liquid nitrogen. The telomerase polymerase reaction was carried out with 24 μL of 1.2x master mix [1x mix contains 1x telomere repeat amplification protocol (TRAP)-buffer (1x TRAP-buffer: 20 mM HEPES-KOH pH 8.3, 1.5 mM MgCl_2_, 63 mM KCl, 1 mM EGTA, 0.1 mg/mL bovine serum albumin (BSA), 0.005% v/v Tween-20), 20 pM of dNTP, 10 pmol of oligonucleotide TS (AATCCGTCGAGCAGAGTT), and 4 μL monocyte or control extract]. The reaction mixture was incubated for 30 min at 25°C. The products were amplified by polymerase chain reaction (PCR) in real time. Thereafter, 1.5 U of Taq-DNA polymerase (Helicon), 10 pmol of oligonucleotide oligonucleotide 5′-GCGCGGCTTACCCTTACCCTTACCCTAACC-3′ (ACX) (CGCGGCTTACCCTTACCCTTACCCTTACC), and Sybr Green I to 0.2x final concentration in the mixture were added in ice (together 2 μL volume). Real-time PCR was carried out on the device CFX-96 for 35 s at 94°C, 35 s at 50°C, and 90 s at 72°C (30 cycles of thermal cycler Mastercycler (Bio-Rad). As a calibration curve, a series of dilutions of cell extracts of telomerase-positive HEK293T cell line (HEK) (15 cells activity was set as 1) and TSR8 [sequence identical to the TS primer extended with eight telomeric repeats AG (GGTTAG)_7_] has been used.

### Statistical Analysis

Data processing and analysis were carried out by using the statistical system SAS 9.1 (SAS Institute, Cary, NC, USA). The results are presented in percentages for qualitative variables and as mean ± standard error of the mean for quantitative variables. Comparison of the signs prevalence in groups was performed by using Fisher’s exact two-tailed test. According to the distribution character, the Mann–Whitney *U*-test or Student’s *t*-test were used for comparing patient groups. The non-parametric Wilcoxon rank-sum test was used in cases when the assumptions, allowing to proceed the paired *t*-test for dependent samples, had not been accomplished. Spearman’s rank correlation coefficient was calculated for statistical description of the relationship between different parameters. Evaluation of the various factors contributing to the magnitude of TA was performed using stepwise regression analysis. Differences were considered statistically significant when *p* < 0.05.

### Study Design

A total of 100 patients aged 35–75 years who met the inclusion and exclusion criteria were included. They were randomized 1:1 to the group treated with atorvastatin 20 mg/day and a control group of patients who did not receive atorvastatin.

All patients were given recommendations for lifestyle modifications (diet and physical activity). After 2 months of atorvastatin treatment, its safety was monitored. At the end of the observation period (12 months) patients were re-examined for parameters included in the study.

## Results

At baseline, the groups did not differ in age, gender, smoking status, level of blood pressure, lipid profile, and other biochemical parameters. Forty-four participants adhered to treatment with atorvastatin; in the group of patients who were not administered atorvastatin, 38 people were re-examined.

The clinical characteristics of the patients in both groups at baseline and after 12 months of treatment are shown in the **Table 1**. In the atorvastatin group, by the end of the year statistically significant reductions in total cholesterol level had been shown (from 6.6 ± 1.1 to 5.8 ± 1.5 mmol/L, *p* = 0.005), as well as reductions in LDL-C (from 4.5 ± 1.0 to 3.9 ± 1.4 mmol/L), triglycerides (1.9 ± 1.1 to 1.5 ± 0.7 mmol/L, *p* = 0.045), ApoB (130.3 ± 36.2 to 96.2 ± 27.7 mg/dL, *p* < 0.001), and increases in ApoA1 (from 173.4 ± 28.8 to 195.1 ± 41.9, *p* = 0.006.). Systolic blood pressure and diastolic blood pressure rates had not changed.

**Table 1:** Characteristics of patients with hypercholesterolemia randomized to the atorvastatin and control groups.

		Atorvastatin (*n* = 44)	Without treatment (*n* = 38)	*p*_1_
Age, years, M ± m	Baseline	59.2 ± 1.2	51.4 ± 1.2	<0.05
Male, % (*n*)	Baseline	34.1 (15)	32.1 (12)	0.848
Smokers, % (*n*)	Baseline	25 (11)	18.4 (7)	0.470
SBP, mm Hg,	Baseline	128.8 ± 2.3	127.5 ± 2.3	0.644
M ± m	12-months	132.5 ± 2.5	124.9 ± 2.3	
	*p*_2_	0.280	0.434	
DBP, mm Hg,	Baseline	80.2 ± 1.6	79.4 ± 1.6	0.729
M ± m	12 months	82.0 ± 1.5	79.2 ± 1.5	
	*p*_2_	0.419	0.928	
TC mmol/L,	Baseline	6.6 ± 0.2	6.1 ± 0.1	0.03
M ± m	12 months	5.8 ± 0.2	6.3 ± 0.2	
	*p*_2_	0.005	0.309	
LDL-C, mmol/L,	Baseline	4.5 ± 0.2	4.4 ± 0.1	0.607
M ± m	12 months	3.9 ± 0.2	4.5 ± 0.2	
	*p*_2_	0.023	0.530	
HDL-C, mmol/L,	Baseline	1.18 ± 0.05	1.21 ± 0.05	0.699
M ± m	12 months	1.27 ± 0.06	1.29 ± 0.06	
	*p*_2_	0.275	0.310	
TG, mmol/L,	Baseline	1.9 ± 0.2	1.2 ± 0.1	<0.05
M ± m	12 months	1.5 ± 0.1	1.3 ± 0.1	
	*p*_2_	0.045	0.844	
ApoA1, mg/dL,	Baseline	173.4 ± 4.3	174.8 ± 4.9	0.819
M ± m	12 months	195.1 ± 6.3	182.8 ± 6.5	
	*p*_2_	0.006	0.334	
ApoB, mg/dL,	Baseline	130.3 ± 5.5	115.7 ± 2.8	0.027
M ± m	12 months	96.2 ± 4.2	104.7 ± 3.3	
	*p*_2_	<0.001	0.017	

*ApoA1, apolipoprotein A1; ApoB, apolipoprotein B; DBP, diastolic blood pressure; HDL-C, high-density lipoprotein cholesterol; LDL-C, low-density lipoprotein cholesterol; M ± m, mean ± standard error of the mean; p_1_, p-value for independent groups; p_2_, p-value for dependent groups; SBP, systolic blood pressure; TC, total cholesterol; TG, triglycerides.*

The main aim of our study was to assess the influence of atorvastatin treatment on TA. Treatment with atorvastatin for 1 year resulted in a statistically significant increase of TA (from 0.46 ± 0.05 to 0.68 ± 0.06, *p* = 0.004) in contrast to the control group, where TA did not change significantly (from 0.67 ± 0.06 to 0.60 ± 0.07, *p* = 0.477). This positive atorvastatin effect on TA was associated with a statistically significant increase in IL-6 and a marginally significant decrease in blood urea. However, there were no statistically significant dynamics of CRP, FBG, or Malondialdehyde values (**Table 2**), although in the atorvastatin group the decline in the proportion of patients with elevated levels of FBG from 31.8% (14 subjects) at baseline to 13.8% (six subjects) after 12 months of treatment (*p* = 0.043) was recorded. In the control group, this parameter has not changed. In order to detect possible determinants of atorvastatin effects on the dynamics of the TA, we conducted the Spearman’s correlation analysis of the association of the TA dynamics and the dynamics of other studied parameters. None of them showed the correlation with the dynamics of the TA (**Table 3**).

**Table 2:** Effects of atorvastatin on the markers of chronic inflammation, oxidative stress, and telomerase activity.

		Atorvastatin (*n* = 44)	Without treatment (*n* = 38)	*p*_1_
CRP, mg/L,	Baseline	5.3 ± 0.9	3.3 ± 0.7	0.1
M ± m	12 months	4.5 ± 0.1	3.0 ± 0.1	
	*p*_2_	0.520	0.721	
FBG, g/L,	Baseline	3.60 ± 0.10	3.26 ± 0.06	0.021
M ± m	12 months	3.51 ± 0.10	3.21 ± 0.07	
	*p*_2_	0.519	0.711	
IL-6, pg/mL,	Baseline	3.64 ± 0.92	5.93 ± 1.91	0.56
M ± m	12 months	6.73 ± 1.04	8.12 ± 3.21	
	*p*_2_	0.021	0.559	
MDA, mcmol/L,	Baseline	3.1 ± 0.1	3.0 ± 0.1	0.596
M ± m	12 months	3.3 ± 0.1	3.4 ± 0.2	
	*p*_2_	0.244	0.086	
Blood urea,	Baseline	6.1 ± 0.2	5.6 ± 0.2	0.139
mmol/L, M ± m	12 months	5.5 ± 0.8	5.3 ± 0.2	
	*p*_2_	0.082	0.37	

*CRP, C-reactive protein; FBG, fibrinogen; IL-6, interleukin 6; MDA, malondialdehyde; M ± m, mean ± standard error of the mean; p_1_, p-value for independent groups, p_2_, p-value for dependent groups.*

**Table 3:** Correlation between the telomerase activity dynamics and the dynamics of the markers of chronic inflammation and oxidative stress.

Indicator	*r*	*p*
ΔTC, mmol/L	0.09	0.421
ΔLDL-C, mmol/L	0.21	0.06
ΔTG, mmol/L	–0.05	0.655
ΔApoA1, mg/dL	0.09	0.438
ΔApoB, g/dL	0.04	0.721
ΔCRP, mg/dL	0.17	0.126
ΔFBG, g/L	0.06	0.592
ΔIL-6, pg/mL	0.01	0.928
ΔBlood urea, mmol/L	0.13	0.244

*Spearman’s correlation coefficients. Δ represents dynamics of studied parameters from baseline to 12 months of atorvastatin therapy; ΔApoA1, apolipoprotein A1 dynamics; ΔApoB, apolipoprotein B dynamics; ΔCRP, C-reactive protein dynamics; ΔFBG, fibrinogen dynamics; ΔIL-6, interleukin-6 dynamics; ΔLDL-C, low-density lipoprotein cholesterol dynamics; ΔTC, total cholesterol dynamics; ΔTG, triglycerides dynamics.*

The independent effect of atorvastatin therapy on the dynamics of TA was confirmed by multifactor linear regression analysis (*p* = 0.05; **Table 4**). Dynamics of all other studied parameters did not show an independent association with TA dynamics.

**Table 4:** Results of multifactor linear regression analysis with the telomerase activity as a dependent variable.

Indicators	β ± SE	*p*	Model *R*^2^
Intercept	0.609 ± 0.405	0.139	
Atorvastatin	0.381 ± 0.192	0.05	
Age	–0.013 ± 0.007	0.083	
ΔCRP	0.008 ± 0.016	0.611	
ΔIL-6	–0.006 ± 0.010	0.559	
ΔFBG	–0.091 ± 0.127	0.410	
ΔBlood urea	–0.052 ± 0.063	0.407	0.1269

*β, regression coefficient; ΔBlood urea, blood urea dynamics; ΔCRP, C-reactive protein dynamics; ΔFBG, fibrinogen dynamics; ΔIL-6, interleukin-6 dynamics; SE, standard error.*

## Discussion

We believe ours to be the first prospective randomized study to demonstrate a positive effect of atorvastatin therapy on TA.

After 1 year of treatment, there was a statistically significant increase in TA in the atorvastatin group compared with the control group, where TA had not significantly changed. The independent impact of statin therapy on the dynamics of TA was confirmed by multifactor regression analysis. According to the results of our study, age had no significant effect on TA dynamics (*p* = 0.083). But even if we accept this *p*-value as significant then we can talk about the negative effect of age on TA dynamics. Nevertheless in the atorvastatin group, where patients were older, increase in TA confirms the activating effect of statin therapy on TA.

It should be noted that there are almost no clinical studies on the effects of statin therapy on TA. Our data are consistent with the results demonstrated by Boccardi et al. (2013a) who showed that statin treatment in people older than 65 years had been associated with higher TA, longer telomeres, and slower telomere shortening, regardless of such factors as age, sex, smoking status, body mass index, the severity of inflammation, glucose level, cholesterol level, and blood pressure. The authors concluded that effects of statin therapy on TL had been mediated by their influence on the TA. In our work, the effect of atorvastatin on TL has not been studied, because the dynamics of TL cannot be evaluated during a 12-month period using our PCR methods. In the available scientific sources, we have found several experimental and clinical studies where a positive effect of statin therapy on TL has been demonstrated. Atorvastatin therapy slows cell aging and prevents telomere shortening in smooth muscle cells, regardless of the degree of oxidative stress reduction (Satoh et al., 2009). Intensive atorvastatin therapy during the year had prevented telomere shortening in contrast with medium-intensive pravastatin therapy in endothelial progenitor cells in patients with CHD (Satoh et al., 2009). Few recent clinical studies confirm a positive effect of statins on TL (Brouilette et al., 2007; Saliques et al., 2011). One of the basic hypotheses is the assumption that statins increase TA. Thus, it can be assumed that statins may be important modulators of TA.

It should be recognized that one of the limitation of the study may be considered the fact that we have not studied the association of TA with apolipoprotein E (ApoE) level and ApoE genetic variants. The potential role of ApoE gene on lipid response to statin treatment and longevity has been investigated by numerous studies (Panza et al., 2007; Zintzaras et al., 2009). It can be assumed that the polymorphism of ApoE gene may determine influence of statins on TA. This issue could be the subject of further research.

Recently, there has been a major focus on finding potential modulators of TA. In experimental studies gene therapy (Boccardi and Herbig, 2012), telomerase-specific promoters (Bernades de Jesus and Blasco, 2012) had been investigated. Recently, it was demonstrated that TA can be influenced by stress and lifestyles (Jacobs et al., 2011; Daubenmier et al., 2012; Lavretsky et al., 2013). Lifestyle intervention, comprising a low-fat diet, regular physical activity, and psycho-emotional stress reduction for 3 months, led to a significant TA increase in mononuclear cells. In another study, it was shown that the Mediterranean diet also led to an increase in TA (Boccardi et al., 2013b). The potential to modulate the TA is of great interest given the importance of telomerase function to cells. As already mentioned, telomerase maintains TL and prevents telomere dysfunction. Since the TL in leukocytes reflects TL in progenitor stem cells, it is these TL and TA that indicate the functional stem progenitor cell state (Oeseburg et al., 2007) and therefore the ability of tissues to repair damage, also in the vessel wall (Boytsov et al., 2013). The relationship between TL and the ability of tissues to repair damages explains the fact that TL and TA can be considered as universal markers of biological aging and aging of the cardiovascular system. This is confirmed by the results of the following studies. The correlation between the short TL in leukocytes and high mortality has been revealed in individuals older than 60 years (Sahin and Depinho, 2010). The association between TL and the duration of healthy life but not between the TL and mortality has been shown in a large cohort study (Njajou et al., 2009). Individuals who had longer telomeres showed better health profile (fewer age-related diseases, better cognitive function, and better lipid composition). The results of several cross-sectional (Satoh et al., 2009; Brouilette et al., 2003) and longitudinal (Brouilette et al., 2007; Farzaneh-Far et al., 2008) studies suggest that there is a relationship between telomere biology and risk of developing and prognosis of CVD. People with shorter telomeres have higher CHD risk. Telomeres are shorter in patients with early CHD onset and myocardial infarction (Brouilette et al., 2007), as well as in patients with carotid atherosclerosis (Nzietchueng et al., 2011). At the same time, TA may be an even more sensitive and modifiable marker of aging and disease than TL. Promising results were obtained both in experimental and clinical studies. TA increase *in vitro* promotes the cells’ lifetime prolongation and the genome stability (Zhu et al., 2000). TA decrease leads to the telomere shortening, regardless of chronological age, and is associated with increased CVD risk (Serrano and Andres, 2004). In healthy women, TA not TL was associated with the main cardiovascular risk factors (Epel et al., 2010).

Telomerase plays an important role in maintaining the normal functioning of the mitochondria, where it can be found besides the nucleus and cytoplasm. This localization under stress conditions may exert a protective effect on mitochondria as well as on the whole cell. Observations in some laboratories showed that telomerase reduced mitochondrial reactive oxygen species production and protected the mitochondrial DNA from damages (Pykhtina et al., 2014).

The second important findings of this study is the observed increase in the level of IL-6 observed during statin therapy. This result may seem surprising, since anti-inflammatory effects of statins are well known, and IL-6 has long been considered a pro-inflammatory cytokine associated with the risk of many age-related diseases, including CVD, and death caused by cardiovascular disorders (Ridker et al., 2000). Moreover, in one study, it has been demonstrated that atorvastatin therapy for 9 months resulted in a decrease of IL-6 (Nawawi et al., 2003). Recently, it was revealed that IL-6 might have both inflammatory and anti-inflammatory effects depending on the type of activated intracellular signaling pathway. The “classical” signaling pathway activation occurs during the joining of IL-6 with the receptor connected with the cell membrane; however, very few cells are capable of expressing the receptors associated with the membrane. Among them are immune cells, hepatocytes, and myocytes. As a result, anti-inflammatory reaction is activated, epithelial cell regeneration after injury occurs, and metabolism in the liver is monitored. Activation of the “trans-signaling” pathway results in binding of IL-6 with the soluble form of the receptor. Vascular endothelium, osteoclasts, and synovial fibroblasts become targets for IL-6. IL-6 pro-inflammatory potential is realized. Such a script can be observed in chronic inflammatory diseases such as Crohn’s disease and rheumatoid arthritis. In this case, STAT3 (signal transducer and activator of transcription 3) mediates this process. STAT3 moves to the nucleus and enhances transcription of many genes, including encoding the formation of acute phase proteins. It can be assumed that the increased expression of IL-6 in healthy individuals is intended to protect the organism from harmful external effects, to restore homeostasis, and to normalize metabolism. This may be confirmed by the results of the work, where the influence of such physiological stimulus as exercise resulted in increased IL-6 levels and improved tissue insulin sensitivity (Pal et al., 2014). It can be assumed that statin therapy leads to the activation of similar mechanisms. It should again be emphasized that in our study, we focused on IL-6 increase in the framework of reference values. In a pathological environment, when IL-6 synthesis regulation is dramatically impaired, cytokine levels increase significantly and chronic inflammation evolves. Increased expression of IL-6 may play a role in the telomerase activation. The possibility of telomerase activation influenced by inflammatory stimuli has been demonstrated in the study by Gizard et al. (2011), which demonstrated that this relationship is realized by the interaction of nuclear factor-kB with the TERT gene promoter. The inhibition of nuclear factor-kB resulted in the cessation of TERT expression. The absence of a statistically significant relationship between the TA dynamics and the IL-6 dynamics in our study may be due to insufficient statistical power of the selection.

We consider that the third important finding of our work is the decline in blood urea level observed during the statin therapy. Our data are consistent with the results of preclinical work, including the study by Maheshwari et al. (2013) which demonstrated the nephroprotective effect of statins, namely the improvement of renal function and reduced urea levels in rats with cisplatin-induced renal dysfunction. According to recent data, urea is recognized as the marker of oxidative stress (D’Apolito et al., 2015). Due to the induction of reactive oxygen species formation in the endothelial cells, mitochondrial urea causes a pro-inflammatory state in endothelial cells, enhancing the formation of advanced glycation end products, activating mechanisms of cellular damage, and causing endoplasmic reticulum stress. The effects of urea are generally similar to the effects of hyperglycemia. Thus, our work, by studying the blood urea as a new marker of oxidative stress, has confirmed the antioxidant properties of atorvastatin.

## Conclusion

The success of recent studies in the field of cellular and vascular aging allows us to identify a clear direction of development for a translational approach to age-related disease prevention. An issue of great interest is the regulation of TA, which has the ability not only to maintain TL, but also to preserve the function of mitochondria and to obtain a beneficial antioxidant and anti-apoptotic effect. Telomerase may play the most important role in the maintenance of optimal cells and tissues function. The preliminary observations may provide grounds to use statins as telomerase activators and effective geroprotectors. In order to reach firmer conclusions, however, we need further extensive studies which would show whether statins could be considered as targeted agents of the prevention of cellular and vascular aging.

## Author Contributions

IS, OT, and DA, contributed substantially to the conception and design of the work, drafting the work, final approval of the version to be published, agreement with all aspects of the work. EP, VP, AK, DK, NK, NS, IO, DS, and DV contributed substantially to the acquisition of the work, drafting the work, final approval of the version to be published, agreement with all aspects of the work. MA and VV contributed substantially to the analysis of data for the work, drafting the work, final approval of the version to be published, agreement with all aspects of the work. SB contributed substantially to the conception of the work, interpretation of data for the work, drafting the work, final approval of the version to be published, agreement with all aspects of the work. ED contributed substantially to the acquisition of the work, drafting the work, final approval of the version to be published, agreement with all aspects of the work.

## Conflict of Interest Statement

The authors declare that the research was conducted in the absence of any commercial or financial relationships that could be construed as a potential conflict of interest.

## Abbreviations

ApoA1: apolipoprotein A1
ApoB: apolipoprotein B
ApoE: apolipoprotein E
CHD: coronary heart disease
CRP: C-reactive protein
CVD: cardiovascular disease
FBG: fibrinogen
IL-6: interleukin-6
TA: telomerase activity
TERC: telomerase RNA component
TERT: telomerase reverse transcriptase
TL: telomere length
